# Hemophagocytosis by acute myeloid leukemia blasts associated to poor clinical outcomes

**DOI:** 10.1002/jha2.392

**Published:** 2022-02-05

**Authors:** Zangbéwendé Guy Ouedraogo, Victoria Cacheux, Louis‐Thomas Dannus, Nathalie Dupre, Lauren Veronese, Benjamin Lebecque

**Affiliations:** ^1^ CNRS Inserm GReD Université Clermont Auvergne Clermont‐Ferrand France; ^2^ Service de Biochimie et Génétique Moléculaire CHU Clermont‐Ferrand Clermont‐Ferrand France; ^3^ Hématologie clinique CHU Clermont‐Ferrand Hôpital Estaing Clermont‐Ferrand France; ^4^ Hématologie Biologique CHU Clermont‐Ferrand Hôpital Estaing Clermont‐Ferrand France; ^5^ Equipe d'Accueil 7453 CHELTER Université Clermont Auvergne CHU Clermont‐Ferrand Hôpital Estaing Clermont‐Ferrand France; ^6^ Cytogénétique médicale CHU Clermont‐Ferrand Hôpital Estaing Clermont‐Ferrand France

**Keywords:** Acute Myeloid Leukemia, AML, Hemophagocytosis

A 74‐year‐old man was referred to the university hospital for fatigue, weight loss, and thrombocytopenia. Initial examination revealed maculopapular lesions on his body.

Subsequent blood tests showed mild normocytic anemia (12.1 g/dL), thrombocytopenia (43 × 10^9^/L), and 3% blast cells among leukocytes (14.17 × 10^9^/L). Coagulation assessment found disseminated intravascular coagulation (DIC). Biopsy of cutaneous maculopapular lesions found a perivascular infiltration by blast cells in the dermis, diagnosing leukemia cutis.

Bone marrow film described infiltration by 76% blast cells. Blast cells were depicted as typical monoblast. Several blast cells showed hemophagocytic activity on erythrocytes (Figure 1A,B) but more rarely on nucleated cells (Figure 1C,D).

**FIGURE 1:**
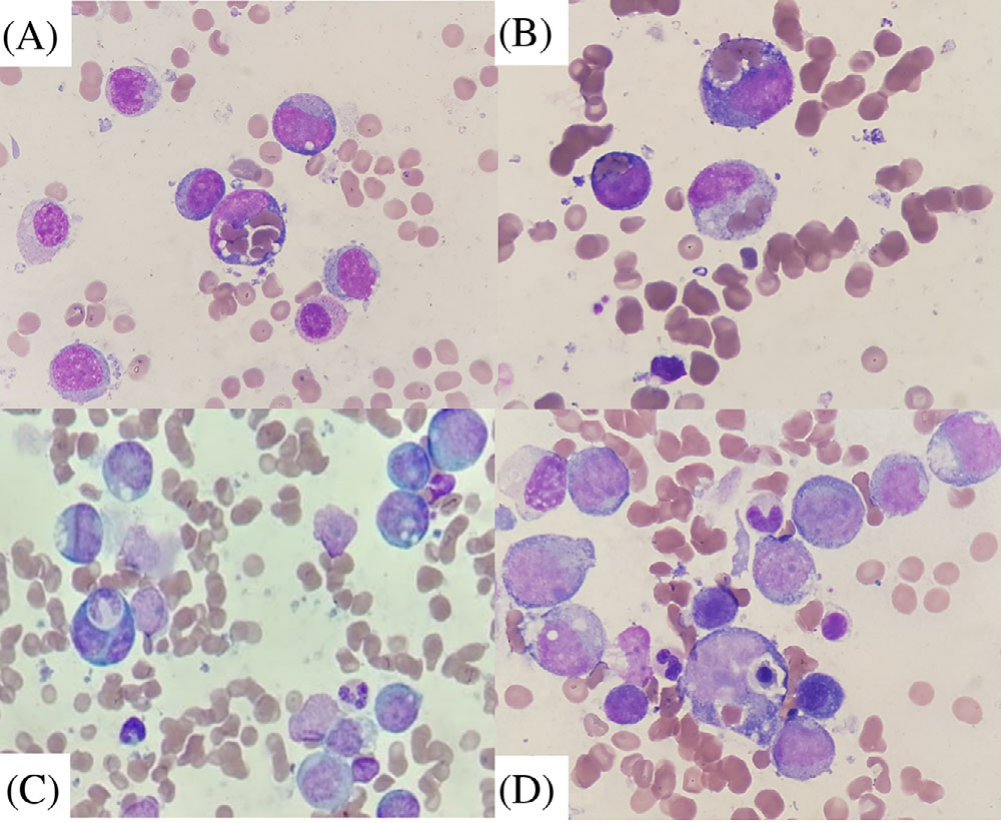


Flow cytometry immunophenotyping revealed an immature cell population accounting for 50% of bone marrow nucleated cells and harboring the following profile: CD4^+^, HLADR^+^, CD13^low^, CD33^+^, CD117^+^ (half of them), CD14^+^ (half of them), and CD34^−^.

Cytogenetic investigations found an isolated loss of the Y chromosome. Next‐Generation Sequencing identified three pathogenic variants: FLT3:c.2503G > T (p.Asp835Tyr) with 13% variant allele frequency (VAF), TET2:c.1304del (p.His435Profs^*^12) with 44% VAF and a rare JAK2 point mutation in exon 19, JAK2:c.2437T > G (p.Tyr813Asp) with 50% VAF.

A diagnosis of acute monocytic/monoblastic leukemia (corresponding to French–American–British M5 of acute myeloid leukemia (AML)) was established according to WHO 2016 classification. Multidisciplinary consultation meeting agreed on chemotherapy treatment using Cytarabin and Venetoclax. Unfortunately, the patient developed a blood transfusion dependency, an aggravation of the cutaneous lesions, and a massive DIC at the start of his treatment and died 19 days later.

Haemophagocytic blasts are described in AML and frequently associated with anomalies of chromosome band 8p11 including t(8;16)(p11;p13) KAT6A::CREBBP, inv(8)(p11q13) KAT6A::NCOA2, t(8;22)(p11;q13) KAT6A::EP300, or t(3;8;17)(q27;p11;q12), or to t(16;21)(p11;q22) with FUS::ERG fusion gene. Our report provides new cytogenetic and molecular insights into this rare entity. Some authors have underlined the recurrence of association of haemophagocytosis by blasts with DIC with worse clinical outcomes, based on analysis of the few reported cases and series. Up to now, this cytological feature is not included in the disease prognostic criteria, but this question should be seriously considered, given the recurrence of disappointing outcomes in clinical cases showing haemophagocytosis on bone marrow smears.

## CONFLICT OF INTEREST

The authors have declared no conflict of interest.

